# Feasibility of Monitoring Health and Well-being in Emerging Adults: Pilot Longitudinal Cohort Study

**DOI:** 10.2196/30027

**Published:** 2022-01-06

**Authors:** Reidar P Lystad, Diana Fajardo Pulido, Lorna Peters, Melissa Johnstone, Louise A Ellis, Jeffrey Braithwaite, Viviana Wuthrich, Janaki Amin, Cate M Cameron, Rebecca J Mitchell

**Affiliations:** 1 Australian Institute of Health Innovation Macquarie University Sydney Australia; 2 Centre for Emotional Health Department of Psychology Macquarie University Sydney Australia; 3 Institute for Social Science Research University of Queensland Brisbane Australia; 4 Department of Health Systems and Populations Macquarie University Sydney Australia; 5 Jamieson Trauma Institute Metro North Hospital and Health Service Queensland Health Brisbane Australia; 6 Centre for Healthcare Transformation Australian Centre for Health Services Innovation Queensland University of Technology Brisbane Australia

**Keywords:** young adult, emerging adulthood, health, well-being, health-related quality of life, feasibility, monitoring, pilot study, longitudinal, cohort, youth, acceptability, survey, quality of life

## Abstract

**Background:**

Emerging adulthood is a distinct segment of an individual’s life course. The defining features of this transitional period include identity exploration, instability, future possibilities, self-focus, and feeling in-between, all of which are thought to affect quality of life, health, and well-being. A longitudinal cohort study with a comprehensive set of measures would be a valuable resource for improving the understanding of the multifaceted elements and unique challenges that contribute to the health and well-being of emerging adults.

**Objective:**

The main aim of this pilot study was to evaluate the feasibility and acceptability of recruiting university graduates to establish a longitudinal cohort study to inform the understanding of emerging adulthood.

**Methods:**

This pilot study was conducted among graduates at a large university. It involved collecting web-based survey data at baseline (ie, graduation) and 12 months post baseline, and linking survey responses to health records from administrative data collections. The feasibility outcome measures of interest included the recruitment rate, response rate, retention rate, data linkage opt-out rate, and availability of linked health records. Descriptive statistics were used to evaluate the representativeness of the sample, completeness of the survey responses, and data linkage characteristics.

**Results:**

Only 2.8% of invited graduates (238/8532) agreed to participate in this pilot cohort study, of whom 59.7% (142/238) responded to the baseline survey. The retention rate between the baseline and follow-up surveys was 69.7% (99/142). The completeness of the surveys was excellent, with the proportion of answered questions in each survey domain ranging from 87.3% to 100% in both the baseline and follow-up surveys. The data linkage opt-out rate was 32.4% (77/238).

**Conclusions:**

The overall recruitment rate was poor, while the completeness of survey responses among respondents ranged from good to excellent. There was reasonable acceptability for conducting data linkage of health records from administrative data collections and survey responses. This pilot study offers insights and recommendations for future research aiming to establish a longitudinal cohort study to investigate health and well-being in emerging adults.

**Trial Registration:**

Australian New Zealand Clinical Trials Registry number ACTRN12618001364268; https://tinyurl.com/teec8wh

**International Registered Report Identifier (IRRID):**

RR2-10.2196/16108

## Introduction

Emerging adulthood is the life stage between adolescence and young adulthood, lasting approximately from ages 18 to 25 years [1,2]. There are many events and factors that can impact the life course of emerging adults [1,2]. Changing health states, different lived experiences, exposure to diverse opportunities, education, and influences from cultural and socioeconomic circumstances can challenge the transition from late adolescence to adulthood, and they have a significant impact on the health and well-being of emerging adults [2-5].

Few longitudinal studies have investigated aspects of health-related quality of life (HRQoL) and well-being in emerging adults. In the United States, 2 university cohorts were established at Harvard University to investigate risk factors for chronic diseases and long-term health in nurses and health professionals [6,7]. In Spain, a prospective university graduate cohort was established at Seguimiento Universidad de Navarra (SUN) to examine dietary habits in the Mediterranean region [8,9]. The scope of the SUN study was subsequently broadened to examine other risk factors and health conditions and expanded to include graduates from 5 other Spanish universities [9]. No longitudinal cohort study comprehensively investigating HRQoL and well-being in emerging adults has been undertaken in Australia. In Australia, Eisenberg et al [10] examined phase transitions of emerging adults, but did not report on any HRQoL or well-being measures. Landstedt et al [11] investigated mental health in a cohort of young Australian adults, but the participants were only asked a single question (ie, “How healthy have you felt mentally during the past 12 months?”), which was not included at baseline.

Conducting a large, prospective longitudinal cohort study of emerging adults using a comprehensive set of measures (eg, physical and mental health, risk factors, life events, resilience, education and employment factors, and social connectedness) would be a unique and valuable resource for improving our understanding of the determinants of healthy and resilient individuals in our society. However, because large, prospective longitudinal cohort studies can be very costly and resource-consuming undertakings, it is essential to first determine the feasibility of conducting such studies.

This pilot study, therefore, aimed to establish the feasibility of recruiting university graduates to establish a large, prospective longitudinal cohort study to inform our understanding of emerging adulthood. Specifically, this pilot study evaluated the following: (1) the feasibility of research methods to recruit university graduates at a large Australian university, including determination of the opt-out rate for data linkage of health records and survey responses; (2) the representativeness of the recruited participants; (3) the ability to obtain baseline survey data, including completion of individual survey instruments; (4) the ability to retain participants and collect follow-up survey data 12 months post baseline, including the completion of individual survey instruments; and (5) opportunities for improving the design of future studies.

## Methods

### Registration

This study was registered with the Australian New Zealand Clinical Trials Registry (ACTRN) on August 14, 2018 (ACTRN12618001364268). The study protocol was published on April 23, 2020 (international registered report identifier: DERR1-10.2196/16108) [12].

### Study Design

This pilot longitudinal cohort study was conducted at Macquarie University in Sydney, Australia. It involved collecting information via web-based surveys (ie, at baseline and 12 months post baseline) and health data record linkage.

### Recruitment

All students graduating from Macquarie University in 2018 (N=8532) were eligible to participate in this study. Macquarie University is a large public university located in a suburban area of Sydney, Australia. At the time of participant recruitment, the university comprised five faculties (ie, Faculty of Arts, Faculty of Business and Economics, Faculty of Human Sciences, Faculty of Medicine and Health Sciences, and Faculty of Science and Engineering), which collectively hosted approximately 45,000 students, including 33,000 undergraduate students, 9000 postgraduate students, and 1500 higher-degree research students.

The graduates were invited to participate via email during the autumn (ie, April) and spring (ie, September) graduation periods. Email invitations included a unique link to a purpose-made website where the graduates were informed about the study before consenting to participate. The initial invitation was followed by 3 reminder emails over a 6-week period. After completing the web-based registration form, participants received an email with an individualized link to the baseline survey.

An incentive to participate was introduced for the second (ie, September) graduation period. The incentive to participate was an entry into a random draw to win 1 of 3 prizes, namely an iPad mini (Apple Inc, first prize) or movie tickets (second and third prizes).

### Survey Data Collection

Surveys were administered via the web-based Qualtrics XM platform (Qualtrics International Inc) at baseline and 12 months post baseline. The baseline and 12-month follow-up surveys comprised the same battery of validated questionnaires and instruments designed to capture data regarding sociodemographic factors, education, employment, job satisfaction, mentoring, self-perceived physical and mental health status, work-life balance, connectedness, resilience, injury, risk behaviors, and life events, as well as social media and technology use. For a detailed overview of the domains and specific questionnaires included in the web-based surveys, see the published study protocol [12].

### Health Record Linkage

Survey responses were linked to personal health information from administrative data collections (ie, ambulance dispatches, emergency department presentations, hospital admissions, cancer registry, and mortality records) in New South Wales (NSW) from April 1, 2018, to 12 months after the completion of the baseline survey. Participants had an opportunity to opt out of having their survey responses linked to their health records during the web-based registration process. The secure health data linkage was conducted by the Centre for Health Record Linkage (CHeReL).

### Outcome Measures

The specific feasibility outcome measures for this pilot study were as follows:

1. Recruitment rate: Calculated as the number of graduates who registered to participate in the pilot study, divided by the total number of graduates.
2. Response rate: Calculated as the number of registered participants who completed the baseline survey, divided by the total number of registered participants.
3. Representativeness: Evaluated by comparing the distribution of graduates and responding participants by faculty and level of qualification.
4. Retention rate: Calculated as the number of participants who completed both the baseline and 12-month follow-up surveys, divided by the number of participants who completed the baseline survey only.
5. Completeness: Calculated as the proportion of missing data for each survey item separately for the baseline and follow-up surveys. For the purpose of this study, survey completeness was categorized as poor (<50%), average (50% to <75%), good (75% to <95%), or excellent (≥95%).
6. Data linkage opt-out rate: Calculated as the number of registered participants who opted out of having their survey responses linked to their health records, divided by the total number of registered participants.
7. Data linkage rate: Calculated as the number of participants who did not opt out of having their survey responses linked to their health records and had one or more health records identified in the Master Linkage Key, divided by the number of registered participants who did not opt out of having their survey responses linked to their health records.
8. Linked record availability: Calculated as the number of available linked health records in each administrative data collection.

### Data Analysis

All data were analyzed using SAS, version 9.4 ( SAS Institute). The recruitment rate, response rate, retention rate, data linkage opt-out rate, data linkage rate, and linked record availability rate were calculated as described above and presented as proportions. Descriptive statistics were used to evaluate the representativeness of the sample and completeness of the baseline and follow-up surveys.

## Results

**Figure 1:**
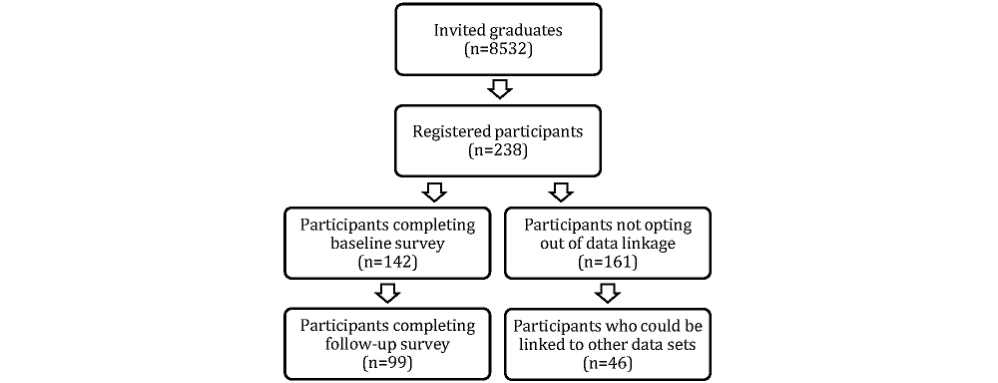
Flow diagram of the selection of the study participants.

A total of 8532 graduates were invited to participate in this pilot study, of whom 238 agreed to participate (Figure 1). This equated to a recruitment rate of 2.8%. Of the 238 graduates who registered to participate in this study, 142 answered the baseline survey. This equated to a response rate of 59.7%. The majority of respondents were female (100/142, 70.4%), single (79/142, 55.6%), and born in Australia (95/142, 66.9%) (Table 1). Compared to the invited graduates, the sample of responding participants was significantly different in its distribution by gender (*P*<.001), but not by level of award (*P*=.14) or graduation time (*P*=.08) (Table 2).

**Table 1:** Demographic characteristics of responding participants.

Characteristic		Respondents (n=142), n (%)
**Gender**		
	Female	100 (70.4)
	Male	41 (28.9)
	Other or unspecified	1 (0.7)
**Marital status**		
	Single	79 (55.6)
	In relationship, not living with partner	5 (3.5)
	In relationship, living with partner	24 (16.9)
	Married	32 (22.5)
	Divorced	2 (1.4)
**Country of birth**		
	Australia	95 (66.9)
	Other specified country	46 (32.4)
	Unspecified	1 (0.7)
**Primary language spoken at home**		
	English	101 (71.1)
	Other specified language	41 (28.9)
**Household income (Aus $)^a^**		
	Less than $50,000 per year	38 (26.8)
	$50,001-$100,000 per year	33 (23.2)
	More than $100,000 per year	52 (36.6)
	Unsure or unspecified	19 (13.4)

^a^Aus $1=US $0.70.

**Table 2:** Representativeness of responding participants.

Characteristic		Graduates (N=8532), n (%)	Respondents (n=142), n (%)	*P* value^a^	
**Gender^b^**					<.001
	Female	4758 (55.8)	100 (70.9)		
	Male	3774 (44.2)	41 (29.1)		
**Level of award^c^**					.14
	Undergraduate	5480 (64.2)	82 (58.2)		
	Postgraduate	3052 (35.8)	59 (41.8)		
**Graduation time**				.08	
	April	5308 (62.2)	78 (54.9)		
	September	3224 (37.8)	64 (45.1)		

^a^Chi-square test for difference in proportions.

^b^Gender was missing for n=1 responding participant, who was omitted from the chi-square test.

^c^Level of award was missing for n=1 responding participant, who was omitted from the chi-square test.

Of the 142 participants who answered the baseline questionnaire, 99 also completed the 12-month follow-up survey. This equated to a retention rate of 69.7%. The completeness of the baseline and 12-month follow-up surveys is shown in Table 3.

Of the 238 graduates who registered to participate in this study, 161 consented to having their survey responses linked to their health records, while 77 opted out of the data linkage component of this study. Thus, the data linkage opt-out rate was 32.4%. Of the 161 registered participants who consented having their survey responses linked to their health records, 46 had used health services and were linked to health records in the CHeReL Master Linkage Key. This equated to a data linkage rate of 28.6%. The most commonly available linked health records were hospital episodes of care and emergency department presentations (Table 4). Unsurprisingly, there were no linked mortality data for this study period. Linkage with cancer registry records was not possible because the most recent cancer registry update preceded the follow-up period for the present study.

**Table 3:** Completeness of survey components at baseline and 12-month follow-up. Completeness was calculated as the number of survey items within a specific survey domain that had missing data, divided by the cross product of the total number of survey items within the specific survey domain and the number of participants not lost to follow-up when the survey was administered.

Domain/Instrument			Baseline (%)		Follow-up (%)
**Sociodemographic factors**					
	Questions about personal status	99.8		99.7	
	Questions about tertiary education	98.8		100.0	
	Questions about employment status	97.9		99.0	
**Working life**					
	Questions about job satisfaction	97.5		96.5	
	Questions about career mentoring	97.3		98.0	
	Role Balance Scale (RBS)	97.1		98.0	
**Health and lifestyle**					
	Questions about physical activity	96.4		96.8	
	Questions about health risk factors	91.5		94.6	
	Short Form Health Survey (SF-12)	95.6		95.8	
	EuroQoL 5-dimension (EQ-5D)	94.8		95.8	
	General Anxiety Disorder scale (GAD-7)	94.5		95.1	
	Social Interaction Anxiety Scale (SIAS-6)	95.1		94.6	
	Kessler Psychological Distress Scale (K10)	94.3		94.6	
	Questions about injury	92.3		94.9	
**Social support and resilience**					
	Questions about social connectedness	93.0		94.9	
	Brief Resilience Scale (BRS)	92.8		94.9	
	Multidimensional Scale of Perceived Social Support (MSPSS)	92.2		94.8	
	Social Readjustment Rating Scale (SRRS)	89.4		93.9	
**Caregiver activities**					
	Questions about caregiver responsibilities and activities	89.4		92.9	
**Social media and technology**					
	Questions about use of social networking sites	89.1		89.5	
	Questions about social media experiences	87.3		89.9	

**Table 4:** Availability of linked records.

Data source	Respondents (n=46), n (%)
NSW^a^ Emergency Department Data Collection	37 (80)
NSW Admitted Patient Data Collection	31 (67)
NSW Ambulance – electronic medical record	6 (13)
NSW Registry of Births, Deaths and Marriages – Death registrations	0 (0)
NSW Cause of Death – Unit Record File	0 (0)
NSW Central Cancer Registry^b^	N/A^c^

^a^NSW: New South Wales.

^b^Linkage not possible because most recent records preceded the follow-up period for this study.

^c^N/A: not applicable. At the time of linkage, the NSW Central Cancer Registry data for the study period were not yet available in the Master Linkage Key.

## Discussion

This pilot study examined the feasibility of recruiting university graduates to establish a longitudinal cohort study to inform our understanding of emerging adulthood. It found that the overall recruitment rate was poor, while the completeness of survey responses among respondents was good to excellent. There was moderate acceptability for conducting data linkage of health records from administrative data collections and survey responses.

### Survey Recruitment and Response Rates

Of the 2.8% (238/8532) of invited graduates who agreed to participate in this pilot cohort study, 59.7% (142/238) responded to the baseline survey. This is considerably lower than the response rate for, for instance, the pilot SUN study (11%) [13] and the Australian arm of the World Health Organization’s World Mental Health Surveys International College Student initiative (7%) [14]. Although the overall recruitment and response rates are disappointing, they are perhaps unsurprising given the general decline in survey participation observed in recent decades [15-17]. Superimposed on this general decline, there is a myriad of factors that may have contributed to the relatively poor recruitment and response rates observed in this pilot cohort study. Dillman’s extension of social exchange theory, the tailored design method, is a theoretical framework that seeks to explain why individuals are motivated to engage in certain social behaviors such as survey participation [18]. This framework suggests that survey response rates depend on reward, cost, and trust. For instance, survey participation is typically more rewarding when participants have a vested interest in the topic [19]. The time spent completing a survey is an important cost consideration for survey participants [19], with longer stated survey length resulting in fewer respondents [20]. In addition to survey length, poor survey structure and design can increase the perceived cost of responding to surveys [19]. In regard to trust, perceived trustworthiness of the organization or institution responsible for administering the survey, confidential use of data, and adequate privacy protections are key elements for reassuring survey participants and improving response rates [19].

It is difficult to determine to which extent each of the abovementioned factors have influenced the recruitment and response rates in this pilot cohort study. Although one might expect recent university graduates to have a vested interest in the topic of health and well-being in emerging adults, perhaps the relative absence of health problems in this age group resulted in a lower interest in the topic and thus lower perceived reward and motivation for participation. For instance, there is evidence suggesting that emerging adults are less motivated by long-term health concerns and lifestyle interventions than older counterparts [21-24]. Additionally, perhaps graduating from university is accompanied by a sense of separation and decreased interest in participating in university-based research surveys. There is also the potential issue of different surveys competing for graduates’ attention and motivation. For instance, Australian university graduates are regularly invited to complete the nationwide Graduate Outcomes Survey, which is one component of the Quality Indicators for Learning and Teaching suite of surveys conducted for the Australian Government Department of Education, Skills and Employment [25]. Competition for attention and motivation becomes a particularly important consideration in the context of emerging adults’ perceived scarcity of time [26]. The graduates invited to participate in this pilot study were informed that it would take approximately 40 minutes to complete each survey (ie, baseline and follow-up). It is conceivable that many potential participants considered the perceived costs of participation in this study to be too high.

Although there is conflicting evidence, some studies have demonstrated that incentives such monetary rewards or lotteries can positively impact response rates [27,28]. In an attempt to improve the recruitment rate in this pilot study, an incentive to participate was introduced for the second (ie, September) graduation period. Consequently, the recruitment rate increased from 2% for the first (ie, April) graduation cohort to 3.8% for the second graduation cohort. The improvement after introducing incentives notwithstanding, the recruitment rate remained disappointingly low. This suggests that the incentives to participate in this pilot study were insufficient to counteract the perceived costs of participation for the vast majority of invited graduates. Perhaps the proliferation and ubiquity of mobile devices in modern society means that the main incentive used in this pilot study (ie, a chance to win an iPad mini) is not perceived as an attractive reward for participating in research.

Previous research has demonstrated that multiple reminders are an effective way to increase response rates [29-31]. However, studies have also shown that the recruitment yield typically declines rapidly with each subsequent reminder [32-34]. This rapidly diminishing marginal return suggests that increasing the number of reminders beyond a small number may not be a cost-effective measure [35]. Hence, Saleh and Bista [19] recommended sending at least 1 reminder, but not more than 3. Furthermore, the desire for increased survey response rates also needs to be balanced with the concern among human research ethics committees that multiple reminders may result in potential survey participants feeling harassed or coerced into participating in the research [36,37].

### Survey Completeness

Survey completeness can be used to refer to 3 different concepts: (1) completeness of the achieved sample with respect to the original one, (2) participation of the respondents throughout all the phases predicted by a research design, and (3) respondents’ propensity to answer all the questions within the questionnaire [29]. The latter 2 concepts were considered in this pilot study. The first of which corresponds to the retention rate between the baseline and follow-up surveys, which was found to be 69.7% (99/142). In regard to the propensity to answer all the questions within a survey, the completeness was excellent, with the proportion of questions answered in each survey domain ranging from 87% to 100% in both the baseline and follow-up surveys. This suggests that the participants did not consider the survey length to be excessive in the pilot study. This is important because previous research has shown that longer survey length can result in both poorer completeness and quality of responses [26].

### Data Linkage

Approximately two-thirds (161/238, 67.6%) of the participants in this pilot study did not opt out of having their survey responses linked to their health records. This suggests that the acceptability of data linkage in the present study was very similar to that in previous Australian studies. For instance, one study reported that 66% of older Australians found it acceptable to have their health data accessed and linked in a registry [38]. Another study of young Australian parents reported, unsurprisingly, that privacy protection was an important consideration for most participants [39]. However, it also was noted that protection measures adopted in best practice health data linkage studies were viewed by most participants as adequate protection for data linkage to proceed without specific individual consent.

### Recommendations

This pilot study offers insights into the feasibility of recruiting recent university graduates to establish a longitudinal cohort study to investigate health and well-being in emerging adults. It is unrealistic to expect a reversal of the general decline in survey response rates. However, it has been suggested that reducing nonresponse rates is less important than minimizing bias in estimates. That is, despite preconceived notions of a good response rate, neither a 5% response rate nor even a 75% response rate necessarily provides unbiased estimates [40,41]. Nonresponse bias occurs when subgroups respond at different rates. In this pilot study, female graduates were more likely to respond to the baseline survey. Overrepresentation of females is common in health-related survey research. Although postsurvey adjustment techniques can be implemented to help reduce nonresponse biases, it is often preferable to prevent nonresponse bias by designing surveys that are more acceptable to the target population in the first instance [42].

In the context of this pilot study, future cohort studies of emerging adults should target populations that are more likely to hold interest in the research. For instance, it might be more useful to target populations at an earlier stage, such as at first enrollment at university or before leaving high school. There are potentially more opportunities for targeted engagement with a student cohort who are commencing postsecondary education. Only a subset of emerging adults undertakes postsecondary education. In Australia, 39.7% of people aged 25 to 34 years had a bachelor’s degree or higher in 2018 [43]. Thus, a cohort of high school graduates offers a less biased sample.

Apart from the choice of specific target populations, future studies should consider the need for personalizing invitations, reducing survey length, crafting surveys that are simple to complete, administering surveys via smartphone apps, and incentivizing participation (eg, through gamification). Lastly, future studies are strongly encouraged to use research codesign to optimize survey parameters. Incorporating the lived experiences of emerging adults into the development and implementation of future research will increase its likelihood of success and impact [44,45].

### Conclusions

The overall recruitment rate was poor, while the completeness of survey responses among respondents was good to excellent. There was reasonable acceptability for conducting data linkage of health records from administrative data collections and survey responses. Future research aiming to establish a longitudinal cohort study to investigate health and well-being in emerging adults should carefully consider the target population as well as how best to obtain an unbiased sample and craft surveys to maximize participation.

## Abbreviations

ACTRN: Australian New Zealand Clinical Trials Registry
CHeReL: Centre for Health Record Linkage
HRQoL: health-related quality of life
NSW: New South Wales
SUN: Seguimiento Universidad de Navarra
